# Neglected rickettsial diseases in Southeast Asia: Twenty-five years of progress in surveillance, diagnostics, and clinical research

**DOI:** 10.1371/journal.pntd.0014318

**Published:** 2026-05-26

**Authors:** Stuart D. Blacksell, Matthew T. Robinson, Kartika Saraswati, Carlo Perrone, Elizabeth M. Batty, Jantana Wongsantichon, Piengchan Sonthayanon, Tri Wangrangsimakul, Ivo Elliott, Paul N. Newton, Nicholas P. J. Day

**Affiliations:** 1 Mahidol Oxford Tropical Medicine Research Unit (MORU), Faculty of Tropical Medicine, Mahidol University, Bangkok, Thailand; 2 Centre for Tropical Medicine and Global Health, Nuffield Department of Medicine, University of Oxford, Oxford, United Kingdom; 3 Lao-Oxford-Mahosot Hospital-Wellcome Trust Research Unit (LOMWRU), Microbiology Laboratory, Mahosot Hospital, Vientiane, Lao People’s Democratic Republic; 4 Saw Swee Hock School of Public Health, National University of Singapore and National University Health System, Singapore, Singapore; 5 Department of Molecular Tropical Medicine and Genetics, Faculty of Tropical Medicine, Mahidol University, Bangkok, Thailand; University of Texas Medical Branch, UNITED STATES OF AMERICA

## Abstract

**Background:**

Rickettsial diseases, including scrub typhus and murine typhus, are major yet persistently under-recognised causes of acute febrile illness in Southeast Asia. Limited diagnostic capacity, ecological complexity, and non-specific clinical presentation have historically contributed to the underestimation of their burden.

**Methods:**

We synthesised 25 years (2001–2025) of integrated epidemiological, clinical, diagnostic, molecular, ecological, and treatment research conducted across Southeast Asia. Evidence from prospective surveillance, hospital-based cohorts, seroepidemiology, molecular characterisation, *in vitro* isolation, genomic analyses, and randomised clinical trials was reviewed to identify convergent findings and policy-relevant lessons.

**Findings:**

Rickettsial infections account for 10%–25% of hospitalised acute febrile illness cases in many endemic settings and are important causes of central nervous system infection, severe disease, and adverse pregnancy outcomes. Diagnostic advances include calibrating IFA and ELISA cut-offs, evaluating rapid diagnostic tests and LAMP assays, developing highly sensitive real-time PCR platforms, and genomic analyses revealing extensive strain diversity. Whole-genome sequencing and multilocus typing demonstrate high recombination and weak geographic structuring of the core genome despite antigenic heterogeneity. Randomised trials confirm doxycycline as first-line therapy for scrub typhus, while azithromycin shows inferior efficacy for murine typhus. Integrated One Health investigations have clarified ecological drivers and vector-host dynamics, and community engagement initiatives have improved awareness in high-risk populations.

**Interpretation:**

Sustained regional investment has transformed rickettsial research from fragmented studies into an integrated surveillance, diagnostic, and translational research framework. This experience provides a transferable model for addressing neglected vector-borne diseases and strengthening febrile illness management in endemic settings. Continued support for laboratory capacity, genomic surveillance, and clinical research is essential to maintain progress and improve regional health system resilience.

## Author summary

Rickettsial diseases, such as scrub typhus and murine typhus, are common yet frequently overlooked causes of fever in Southeast Asia. Because symptoms resemble those of many other infections, these diseases are often misdiagnosed or treated late, leading to preventable complications. Over the past 25 years, collaborative research across Thailand, Laos, Vietnam, and neighbouring countries has improved understanding of how these infections spread, how they can be diagnosed more accurately, and how best to treat them. Studies have shown that rickettsial infections account for a substantial proportion of hospitalised fever cases and can cause severe disease, especially in children and pregnant women. Diagnostic improvements, including calibrated serological tests and molecular assays, have enhanced detection and surveillance. Genomic studies have revealed high genetic diversity, which may explain why single-antigen tests perform inconsistently. Clinical trials have strengthened treatment recommendations, confirming doxycycline as an effective therapy for scrub typhus. This review summarises the lessons learned from a quarter-century of research and highlights how sustained regional investment can build durable health system capacity to manage neglected infectious diseases.

## 1. Introduction

Rickettsial diseases are caused by vector-borne, Gram-negative, intracellular bacteria belonging to the order *Rickettsiales* [1]. The primary antigenic groups of human interest are the Scrub Typhus Group (STG), mainly caused by *Orientia tsutsugamushi*; the Spotted Fever Group (SFG), which includes over 20 *Rickettsia* species; and the Typhus Group (TG), which includes murine typhus (*Rickettsia typhi*) and epidemic typhus (*Rickettsia prowazekii*) [2,3]. These groups are distinguished based on their vectors, antigenic properties, and clinical manifestations [4]. Despite their high incidence across Asia and emerging presence in other regions, these zoonotic infections are frequently misdiagnosed or overlooked due to their nonspecific clinical presentation and diagnostic limitations [4]. Neglect persists despite scrub typhus causing especially substantial morbidity and mortality across diverse populations, including children, pregnant women, and rural communities.

Rickettsial diseases have been recognised in Asia for more than a century, with early descriptions of scrub typhus in Japan in the late nineteenth century and subsequent identification across Southeast Asia. During the Second World War, scrub typhus caused substantial morbidity among Allied forces, prompting intensive investigations by military research groups in the Maldives, India, and Myanmar that helped clarify the transmission, clinical features, and vector ecology of scrub typhus and other typhus group infections [5–7]. Post-war research units, including those in Kuala Lumpur, further advanced diagnostic approaches and therapeutic evaluation, contributing to the adoption of chloramphenicol as an effective treatment before the tetracycline era [6,8]. Despite these advances, civilian research activity remained limited for decades. Until approximately 25 years ago, rickettsioses in much of Southeast Asia were under-recognised outside military or outbreak settings, with diagnostic capacity largely confined to specialised centres, far from patients.

This review synthesises epidemiological, clinical, diagnostic, and ecological evidence on rickettsial diseases generated over the past two decades across Southeast Asia. Evidence is drawn from a combination of long-running research collaborations, national hospital-based studies, community serosurveys, pregnancy and paediatric cohorts, and One Health investigations conducted by multiple academic, governmental, and non-governmental groups across the region. While several insights derive from established research programmes with sustained capacity for surveillance and diagnostics, findings are interpreted alongside independent studies from diverse geographic and health-system contexts to identify convergent regional patterns and inform generalisable policy and practice recommendations.

## 2. Methods

This article presents a narrative synthesis of rickettsial disease research conducted in Southeast Asia, including Thailand, Lao PDR (Laos), Cambodia, Myanmar, Malaysia, Indonesia, and Vietnam, between 2001 and 2025. The review integrates published literature with findings from long-term surveillance programmes conducted by the authors and collaborating institutions in Southeast Asia. Relevant studies were identified through targeted searches of PubMed, Web of Science, and institutional project outputs, combined with expert knowledge of long-running surveillance programmes in the region. This review was conducted as a narrative synthesis and does not follow formal systematic or scoping review methodology. Study selection was guided by targeted literature searches and the authors’ knowledge of long-standing research programmes in the region.

## 3. Rickettsial disease burden and epidemiology in Southeast Asia

### 3.1 Overview: Burden, heterogeneity, and under-recognition

The epidemiology of rickettsial diseases in Southeast Asia is highly heterogeneous, reflecting variation in ecological conditions, land use, vector distributions, and patterns of human behaviour. Rural and peri-urban areas characterised by agriculture, forest fringes, and seasonal labour are consistently associated with a higher risk for scrub typhus. Across Thailand [9–18], Laos [19–25], Cambodia [26–28], Myanmar [29–31], Malaysia [32–34], Indonesia [35–38], Vietnam [39–47] (Table 1), reported scrub typhus seroprevalence varies widely depending on study design, the population sampled, and the diagnostic methods used; from less than 1% to approximately 15% in Malaysia, Indonesia, and some urban or low-risk Thai settings to 30–60% in rural Thailand, Myanmar and Vietnam. Urban transmission of murine typhus remains an important but often overlooked contributor to the burden of febrile illness [19,22]. This heterogeneity underscores that rickettsial disease burden cannot be inferred from single-site studies or narrow ecological assumptions and highlights the limitations of surveillance systems that rely on pathogen-specific testing or passive reporting.

**Table 1:** Summary of key studies demonstrating the substantial, heterogeneous, and often under-recognised burden of rickettsial diseases across Asia, highlighting ecological, social, and health-system drivers of transmission and detection.

Country/setting	Study/reference(s)	Study sites/collaborators	Key findings (including quantitative results)	Implications
Laos	Hospital-based febrile illness studies [19,22]; CNS infection studies [20,21];vector and ecological studies [23–25]	LOMWRU, Mahosot Hospital (Vientiane)	Scrub and murine typhus account for up to 25% of febrile hospital admissions, with scrub typhus a leading non-malarial cause of undifferentiated febrile illness (UFI). Rickettsioses comprise up to one-third of central nervous system (CNS) infections in some cohorts. *R. asembonensis* is detected in more than 30% of dog fleas, and *O. tsutsugamushi* is found in chiggers and small mammals. These infections are strongly associated with land-use change and climate variability.	Demonstrates high, under-recognised burden with clear ecological and zoonotic drivers
Thailand (Thai–Myanmar border)	Pregnancy cohort studies [9]	SMRU	Scrub typhus identified as the leading cause of febrile illness in pregnancy; incidence estimated at 6–8 per 1,000 pregnancies with significant maternal and perinatal complications	Highlights severe outcomes in a high-risk population often excluded from surveillance
Thailand (Northern Thailand)	Adult and paediatric UFI studies [10,11]; strain characterisation [12]; landscape and socioeconomic studies [15,16]	Chiang Rai; Nan province	Scrub typhus confirmed as the dominant bacterial cause of adult UFI (15%–25%) and a frequent cause of severe paediatric illness; seroprevalence in rural communities ranging from 20% to 50%; identification of novel *O. tsutsugamushi* strains; exposure shaped by land use, elevation, and socioeconomic factors	Shows heterogeneous risk driven by ecological and social determinants
Thailand (Urban Bangkok)	Urban zoonotic studies [13,14]	Bangkok	Rickettsial pathogens detected in 10%–30% of sampled dogs, cats, and small mammals in public parks; evidence of sustained urban transmission cycles	Challenges the assumption of rickettsioses as purely rural diseases
Cambodia	Paediatric hospital surveillance [26]	COMRU, Siem Reap	Scrub and murine typhus together accounted for 20%–30% of paediatric febrile hospital admissions; limited routine diagnostic capacity	Indicates substantial paediatric burden and diagnostic gaps
Myanmar	Urban and peri-urban exposure studies [29–31]	Urban-linked sites	High seroprevalence of scrub and murine typhus antibodies (30–60%) among adolescents and adults; frequent misclassification as dengue or undifferentiated fever	Suggests under-recognised urban and peri-urban transmission
Vietnam	Hospital-based UFI and CNS infection studies [41,44,45]; regional serological and epidemiological studies [39,42,43]; historical studies [46]	Multiple provincial hospitals	Scrub and murine typhus responsible for 10%–40% of adult UFI in hospital studies; seroprevalence estimates ranging from 15% to 60% depending on region; rickettsioses identified as significant contributors to CNS infections; long-standing endemicity documented over five decades	Demonstrates sustained transmission and highlights persistent surveillance and diagnostic limitations
Indonesia	Archived sample analyses and mapping studies [35,36,38,63,164,165]; regional ecological analyses [62]	Multi-island studies	Seroprevalence of scrub typhus antibodies ranging from 0.36% to 20% across islands; long-standing endemicity masked by diagnostic limitations; seroincidence modelling and spatial mapping identified high-risk zones; SFG rickettsioses shown to be ecologically embedded zoonoses; *Rickettsia* is a leading cause of hospitalised acute febrile illness	Illustrates long-standing, under-detected endemicity and value of novel analytical approaches
Malaysia	Case report and hospital studies [32–34]	Sabah; Peninsular Malaysia	Murine typhus identified in 5%–15% of hospitalised febrile patients; frequent misdiagnosis as leptospirosis or dengue; occupational exposure documented	Exposes persistent diagnostic blind spots

### 3.2 Magnitude of burden: Incidence, seroprevalence, and hidden transmission

Taken together, studies demonstrate that rickettsial infections are among the most common yet persistently neglected causes of febrile illness across Southeast Asia. Clinical studies identify rickettsioses as leading causes of fever [19,22,48], while serological and ecological investigations reveal far greater population exposure than recognised by routine passive surveillance [49–53]. This evidence provides a strong foundation for integrating rickettsial diagnostics into febrile illness algorithms and prioritising rickettsioses within regional public health policy.

Across Southeast Asia, rickettsial infections have consistently emerged as major causes of acute febrile illness in both hospitalised and community populations. In Thailand, early prospective studies from the Thai-Myanmar border demonstrated that arthropod-borne infections, particularly scrub typhus and murine typhus, were the leading causes of fever in pregnancy, accounting for a substantial proportion of non-malarial febrile episodes and contributing to adverse maternal and neonatal outcomes [9]. Subsequent studies in northern Thailand confirmed that rickettsial diseases were among the most common aetiologies of acute undifferentiated fever in both adults and children, frequently rivalling or exceeding dengue in incidence [10–12]. Paediatric cohorts hospitalised with scrub typhus showed significant morbidity, including organ dysfunction, and mortality, underscoring that these infections are not benign and that delayed recognition translates directly into avoidable harm [11].

Across Thailand, clinical, laboratory, and radiological studies have established scrub typhus as a frequent and often severe cause of hospitalised febrile illness. Among 130 Thai patients, pulmonary involvement was common, with interstitial infiltrates and consolidation frequently observed, demonstrating that scrub typhus is a systemic infection with substantial respiratory morbidity [54]. Another study described the development of a simple risk-scoring system to predict scrub typhus severity using seven years of retrospective data from two university-affiliated hospitals in northern Thailand. The score incorporates readily available clinical and laboratory parameters to stratify patients into non-severe, severe, and fatal risk categories with good predictive accuracy, although external validation is required before routine clinical use [55]. Subsequent studies in northeastern Thailand identified specific clinical features, including the presence of eschar, electrocardiographic abnormalities, and markers of organ dysfunction, as strong predictors of fatal outcome, underscoring the importance of early recognition and treatment in endemic settings [56]. Despite consistent and mounting evidence of the importance of rickettsial diseases, even in endemic settings, specific treatment is seldom included in empiric regimens [57].

In Thailand, seroprevalence in rural communities, hill-tribe populations, and along landscape gradients frequently exceeds 20%–30% in adults, with strong associations with agricultural activity, proximity to forests, and occupational risk [15,16]. A nationwide seroepidemiological study assessed the IgG seroprevalence of STG, TG, and SFG rickettsiae among Royal Thai Army recruits using archived sera from 2007 to 2008 and 2012 [58]. The findings demonstrate widespread rickettsial exposure in Thailand, with STG showing the highest and significantly increasing seroprevalence over time, particularly in southern regions, highlighting the endemic nature of rickettsioses and underscoring the need for improved awareness, surveillance, and prevention efforts [58,59].

In Myanmar, hospital- and community-based studies quantified both seroprevalence and incidence, confirming rickettsial infections as common causes of fever and indicating far broader exposure than is captured by routine case detection [29–31,58].

In Laos, prospective studies have shown that *O. tsutsugamushi*, spotted fever group *Rickettsia*, and *R. typhi* together account for a substantial fraction of hospitalised febrile and central nervous system (CNS) infections [19–22,60], and are among the leading causes of acute undifferentiated fever [19,22]. Strikingly, doxycycline-responsive infections such as scrub typhus, murine typhus, and leptospirosis were more common causes of CNS infections than ‘conventional’ bacterial pathogens, suggesting that doxycycline should be added to third-generation cephalosporins in the empirical treatment of CNS infections.

Longitudinal analyses link incidence to land-use change, seasonality, and climate variability [24]; however, even within countries, seasonality and the relative importance of climatic drivers (e.g., temperature and relative humidity) can vary [61]. Across multiple countries, transmission is consistently associated with agricultural intensification and environmental disruption [24,35,36,38,61,62].

Studies from Vietnam show that scrub typhus and murine typhus are major causes of undifferentiated febrile illness, together accounting for 10%–40% of adult and paediatric cases in hospital-based studies, and are also important contributors to central nervous system infections [41,44,45]. Serological surveys across multiple regions demonstrate high population exposure, with seroprevalence varying widely by geography and reflecting heterogeneous ecological risk [39,42,43]. Historical and contemporary evidence indicate sustained endemic transmission over several decades, highlighting persistent diagnostic gaps and suggesting that the actual burden of rickettsial disease in Vietnam remains substantially underestimated [46].

Compared with scrub typhus and murine typhus, relatively little research has been performed on SFG rickettsioses in Asia. A regional synthesis [62] demonstrated that SFG *Rickettsia* spp. are widely distributed across diverse ecological settings in Asia, with transmission strongly associated with tick vectors, land-use patterns, wildlife hosts, and climatic drivers, yet human disease remains under-recognised and under-diagnosed due to limited molecular confirmation and surveillance capacity. Importantly, the review found no robust evidence for the presence of epidemic typhus (*R. prowazekii*) or rickettsialpox (*R. akari)* in Southeast Asia, although few investigations have specifically targeted these pathogens. The apparent absence of reported cases, therefore, likely reflects limited focussed surveillance rather than confirmed absence, highlighting the need for expanded molecular diagnostics, vector studies, and ecological investigations to better define the burden and distribution of SFG rickettsioses in the region.

### 3.3 Urban and peri-urban rickettsial transmission

Importantly, rickettsial transmission is not confined to rural or forest-fringe settings. Urban and peri-urban investigations in Bangkok, Yangon, Vientiane, Peninsular Malaysia, and Indonesia identify murine typhus and spotted fever group rickettsioses as frequent but under-recognised causes of febrile illness, often misdiagnosed as dengue or leptospirosis [16,24,29–34,63]. Molecular detection of *O. tsutsugamushi* and significant prevalence of anti-*O. tsutsugamushi* IgG in Vientiane may reflect urban transmission in parks and farmland within the city, or movement of people between the city and surrounding rural areas [64]. Detection of rickettsial pathogens in domestic animals, small mammals, and ectoparasites within urban environments further supports sustained urban transmission cycles [13,14]. Complementing human data, One Health investigations document the widespread circulation of rickettsiae in animal reservoirs and vectors across the region, including in urban and peri-urban environments [13,14,23,25]. Comparable patterns are reported across Malaysia and Indonesia [32–36,62].

Outbreak investigations in northern and central Thailand documented spatially clustered scrub typhus transmission linked to local ecological conditions, including peri-urban and industrial landscapes, demonstrating that disease risk extends beyond traditional endemic zones [65,66]. Urban and peri-urban transmission further challenges the assumption that rickettsioses, except murine typhus, are confined to rural areas, as molecular detection of *R. felis* and *R. felis-*like DNA in human and dog blood samples in Bangkok confirms their circulation in metropolitan environments [67].

Synthesising these findings, rickettsioses are increasingly recognised as major yet under-appreciated causes of febrile illness in both endemic populations and travellers, with frequent misdiagnosis and delayed therapy persisting, particularly during periods of health-system strain [68].

### 3.4 Populations at highest risk

Rickettsial diseases in Southeast Asia disproportionately affect populations already vulnerable to health inequities. In endemic settings, scrub typhus and other rickettsial infections are most common among agricultural workers and rural communities, where occupational and environmental exposure to vector-infested habitats increases infection risk and limited access to diagnostics and care delays appropriate treatment. Studies in rural Thailand demonstrate that socio-demographic factors, such as occupation and local ecological conditions, significantly influence human exposure to *O. tsutsugamushi*, highlighting that livelihoods tied to agriculture and domestic animal ownership are associated with a greater risk of exposure [15].

Pregnant women, older adults, and children represent particularly high-risk groups for severe outcomes [69]. Although data on typhus in pregnancy remains limited [70,71], cohort and case-series analyses from Asia show that scrub typhus and murine typhus during pregnancy are associated with substantial rates of adverse maternal and foetal outcomes, including miscarriage, stillbirth, preterm delivery, and growth restriction, particularly when diagnosis and treatment are delayed [72]. Paediatric infections likewise frequently present with severe clinical manifestations when diagnosis is delayed, and rickettsial diseases are increasingly recognised as under-appreciated contributors to acute febrile illness across local communities in the region [34]. These patterns reinforce the need for febrile illness strategies that explicitly consider vulnerable populations, incorporating targeted risk assessment and accessible diagnostics rather than relying solely on broad syndromic approaches that may miss or delay identification of rickettsial infections, especially in high-risk groups.

Older adults represent an additional under-characterised high-risk group. Although data remain limited in Southeast Asia, increasing age is associated with a greater risk of severe disease, organ dysfunction, and mortality in scrub typhus, likely reflecting comorbidities and delayed healthcare access. Improved age-stratified surveillance and clinical studies are needed to better define disease burden and optimise management in ageing populations.

In children, multiple cohort studies from Southeast Asia demonstrate that scrub typhus is a significant cause of hospitalised febrile illness, with potential for severe complications including organ dysfunction and death when diagnosis is delayed. These findings highlight the need for paediatric-specific diagnostic algorithms and earlier empiric treatment strategies in endemic settings.

### 3.5 Implications for health systems and surveillance

Taken together, these epidemiological patterns highlight a persistent mismatch between the actual burden of rickettsial diseases and their recognition within health systems in Southeast Asia (Panel 1). Although scrub typhus, murine typhus, and related infections are major causes of undifferentiated febrile illness, their incidence is consistently underestimated due to under-recognition, limited diagnostic capacity, and frequent misclassification in routine clinical care [2,68]. Most patients present to district-level facilities where laboratory confirmation is rarely available, contributing to delayed or incorrect diagnoses. Marked regional heterogeneity in seroprevalence and case detection further indicates that passive reporting fails to capture key transmission dynamics [68]. Thailand is the only Southeast Asian country where scrub typhus is notifiable through a national disease surveillance system. The heterogeneous distribution of cases reported, with the highest numbers in the north, northeast, and south, is described in other countries with surveillance systems (China, Taiwan, South Korea, and Japan). Even at the scale of a single Thai province, Chiang Rai, case distribution shows marked heterogeneity, apparently influenced by elevation, population size, habitat complexity, and landcover diversity [61,73]. In this context, sentinel surveillance approaches, including hospital-based fever studies and complementary serological, animal, or environmental monitoring, provide a feasible and cost-effective means of identifying under-recognised transmission and informing public health priorities [74,75].

## 4. Diagnosis and pathogen characterisation

Diagnostic uncertainty has long hindered recognition, treatment, and surveillance of rickettsial infections in Southeast Asia. Through influential studies (Table 2) and collaborations, diagnostics are gradually moving from fragmented, ad hoc systems to a more standardised, evidence-based system. The mainstay of laboratory diagnosis remains serology, which ideally requires paired acute and convalescent samples to assess antibody titre rises. This limits the utility of serological diagnosis in the acute setting. For Southeast Asian health systems, these findings show that relying solely on a rapid diagnostic test based on serology is inadequate for managing febrile illness; calibrated (and ideally combined) serological and molecular diagnostics are crucial for surveillance, outbreak detection, and policy [2]. These advances shape national guidelines, surveillance, and case management protocols.

**Table 2:** Evaluations of diagnostic assays for scrub and murine typhus.

Diagnostic method	Target/principle	Diagnostic performance (reported values)	Key strengths	Key limitations	Studies
IFA (Indirect Immunofluorescence Assay)	IgM/IgG antibodies	Sensitivity typically 60%–90% depending on titre cut-off and timing; specificity >90% when appropriate thresholds applied; substantial inter- and intra-reader variability	Widely used reference serological method; quantitative	Poor early sensitivity; lack of standardised cut-offs; reader-dependent; requires paired samples for maximum utility	[52,53,76,78]
Finger-prick IFA	IgM/IgG antibodies (capillary blood)	Sensitivity and specificity comparable to venous blood IFA (no significant loss of accuracy reported)	Enables decentralised sampling; field-appropriate	Requires fluorescence microscopy and trained readers; limited utility as an acute diagnostic	[77]
ELISA (IgM/IgG)	IgM/IgG antibodies (recombinant or whole-cell antigens)	Sensitivity 70%–95%, specificity 80–98% when site-specific cut-offs applied; multivalent recombinant ELISAs significantly outperform single-strain assays	More reproducible than IFA; amenable to automation; scalable	Timing-dependent; cut-offs not transferable without local calibration; limited utility as an acute diagnostic	[80,89–95]
Rapid Diagnostic Tests (RDTs)	IgM (± IgG) antibodies	Sensitivity ~50%–90% across studies; specificity variable (~70%–95%); markedly reduced sensitivity early in illness	Fast; point-of-care; minimal infrastructure	Highly inconsistent performance; unreliable as standalone test	[82–86]
LAMP (Loop-mediated isothermal amplification)	Pathogen DNA	Sensitivity generally 70%–90% in acute infection; specificity >95%; detects infection prior to seroconversion	Rapid; low-cost; field-deployable; suitable for district hospitals	Pathogen-specific; lower sensitivity than qPCR; limited commercial availability	[87,108,110,166]
qPCR (e.g. groEL-, 47-kDa–targeted)	Pathogen DNA	Analytical sensitivity often <10 copies/reaction; specificity >95%; highest clinical sensitivity during early illness	Detects acute infection before antibody development; reference molecular method	Requires molecular laboratory; sensitivity declines after antibiotic initiation	[100,101,104,109]
Multiplex PCR	*Orientia* and *Rickettsia* spp. DNA	Analytical sensitivity comparable to single-plex PCR (detection limits 1–10 copies/µL reported); high specificity	Enables simultaneous detection of co-endemic pathogens; supports syndromic diagnosis	Laboratory-based; limited routine availability	[102,103]

### 4.1 Standardisation and development of serological methods

The lack of standardised locally appropriate indirect immunofluorescence assay (IFA) cut-offs and the intrinsic limitations of antibody-based detection for scrub typhus, and probably other rickettsioses, lead to inconsistencies in case definitions [52,53], resulting in misclassification, false positives, and overdiagnosis of co-infections [76]. These issues hinder cross-study comparisons and accurate burden estimates across regions. Geographically appropriate serological cut-offs are vital for reliable disease estimates, reporting, and inclusion in fever algorithms. Field evaluations demonstrated that finger-prick blood spots can reliably replace venous blood for IFA testing, making it feasible in low-resource settings [77]. Variability in IFA slide interpretation highlighted the need for standardised training and quality control [78]. Precise serological calibration proved clinically crucial in paediatric cases [79]. Collaborations in Thailand led to the incorporation of recombinant antigens for the 56-kDa scrub typhus protein into ELISA protocols, thereby improving accuracy and standardisation [80]. These efforts advance a more consistent, accessible, and clinically relevant diagnostic framework for rickettsial diseases.

Early diagnostic approaches for scrub typhus were constrained by reliance on single-strain antigens, limiting sensitivity in settings characterised by high strain diversity. Building on accumulating evidence of genetic and antigenic heterogeneity, Chao et al. demonstrated that ELISA platforms incorporating recombinant proteins from multiple *O. tsutsugamushi* strains substantially improved diagnostic accuracy in endemic populations [81]. This work provided practical confirmation that multivalent antigen design could overcome regional strain variability and anticipated later genomic and antigenic geographic variation findings. It marked a shift away from narrowly targeted assays toward regionally robust serological tools suitable for both surveillance and clinical use.

### 4.2 Evaluation of commercial and in-house rapid diagnostic tests

Coordinated efforts in Thailand and Laos translated diagnostic principles into rapid, field-deployable formats. Broadly reactive lateral flow assays detecting IgM and IgG against *O. tsutsugamushi* demonstrated improved sensitivity across diverse clinical presentations, but field evaluations revealed substantial variability in performance, underscoring the need for local validation of point-of-care tests; similarly, in endemic settings, about 30% of RDT-confirmed cases were found to be false positives [82–84]. Multiple evaluations of IgM-based rapid diagnostic tests (RDTs) for scrub typhus and murine typhus showed wide variability in sensitivity (24%–96%) and specificity (73%–100%), limiting their reliability for early clinical decision-making [85,86]. However, combining antibody-based RDTs with molecular tools such as loop-mediated isothermal amplification (LAMP) improved detection during the acute phase of infection [87].

A systematic review and meta-analysis confirmed the inconsistent performance of commercially available scrub typhus RDTs and reinforced the need for validated point-of-care diagnostics tailored to local strain diversity and setting [84]. Alternative formats such as dot-ELISA using recombinant 56-kDa antigens achieved high diagnostic accuracy while remaining technically simple, demonstrating that performance gains depend on antigen selection rather than assay complexity [88]. Collectively, experience across Southeast Asia shows that no single rapid test is sufficient, that antibody-based assays have limited utility early in infection, and that local validation is essential, highlighting the need for integrated diagnostic strategies for both clinical care and surveillance.

### 4.3 Evaluation of commercial and in-house ELISAs

Studies validated site-specific ELISA cut-offs, enabling more accurate diagnosis in Thailand, Laos, and Bangladesh using the commercial InBios ELISA [89–91]. Building on this work, optimal cut-off values were identified for the de facto in-house scrub typhus IgM and IgG ELISA developed by the US Navy’s Naval Medical Research Center (Silver Spring, Maryland, USA) [92–94], thereby reducing diagnostic misclassification and improving the accuracy of surveillance and case management. Subsequent systematic review synthesised evidence from more than 20 regional studies, guiding the use of scrub typhus ELISA thresholds and supporting harmonised recommendations across laboratories [95].

### 4.4 Redefining diagnostic thresholds

Improving diagnostic cut-offs for rickettsial and other neglected infections addresses the persistent challenge posed by the absence of a reliable gold standard. Bayesian latent class modelling was employed to assess diagnostic accuracy in the absence of a reference standard, enabling the derivation of thresholds applicable to Chiang Rai, northern Thailand [93,96,97]. This work was extended through investigations of humoral immunity in murine typhus, resulting in more accurate, location-specific IFA cut-offs across different disease phases [98]. In addition, the first systematic review of murine typhus IFA thresholds was conducted, providing an evidence base to support diagnostic standardisation [99]. Collectively, these efforts contributed to a shift toward harmonised, evidence-based diagnostic approaches adopted by regional laboratories, helping address long-standing challenges such as assay variability and operator dependence characteristic of many neglected tropical diseases.

### 4.5 Molecular diagnostics and other innovations

Molecular assays rapidly became central to rickettsial diagnostics in Southeast Asia. Early work in rural Thailand demonstrated that PCR for *O. tsutsugamushi* could be implemented in low-resource laboratories, thereby improving early diagnosis and clinical management [100]. Subsequent optimisation of highly sensitive quantitative PCR assays targeting the *groEL* and 47-kDa genes improved diagnostic performance for local strains [101], and the development of multiplex PCR platforms enabled simultaneous detection and differentiation of *Orientia* and *Rickettsia* species while maintaining high analytical sensitivity [102]. These approaches supported syndromic diagnosis in settings where multiple rickettsial pathogens co-circulate, and serology is often uninformative.

Further refinement introduced multiplex real-time PCR assays incorporating internal host-response controls, thereby improving reliability during the acute, pre-seroconversion phase of illness [103]. Today, the 47-kDa real-time PCR assay developed by the US Navy remains the most widely used molecular test for the diagnosis of scrub typhus [104]. PCR assays targeting the 16S rRNA gene have also demonstrated high sensitivity and specificity with the additional benefit of detecting a broader range of strains [105]. Eschar swab PCR, performed only for patients with eschars, has become an excellent diagnostic tool for scrub typhus, offering high sensitivity and specificity while being minimally invasive in clinical and field settings compared to traditional blood-based methods [106,107]. In parallel, LAMP-based assays provided rapid, affordable diagnostic options suitable for district hospitals, with prospective studies confirming their clinical utility [87,108–110]. Together, these advances established a layered diagnostic framework that integrates molecular assays, rapid tests, and serology, which is now embedded in febrile illness guidelines across Southeast Asia [74]. Some authors have also targeted repeated regions of the *O. tsutsugamushi* genome (the *traD* gene), substantially increasing PCR sensitivity compared to a single-gene target [111,112]. More recently, recombinase-assisted amplification and CRISPR/Cas assays have been developed and tested, with promising results [113–115]. These technologies could simplify the use of molecular methods in peripheral or field settings.

These advances collectively reframed rickettsial diagnostics in Southeast Asia as a layered system rather than a single test solution. Serology, rapid tests, and molecular assays each serve distinct roles across the clinical and surveillance continuum. Experience demonstrates that effective rickettsial diagnosis depends not on the wholesale adoption of any one platform, but on calibrated integration of tools matched to epidemiology, clinical need, and laboratory capacity. This systems-based approach has direct implications for national febrile illness algorithms, laboratory referral pathways, and regional preparedness for both endemic and emerging vector-borne infections.

## 5. *In vitro* isolation of *Orientia* and *Rickettsia* spp.

Effective diagnostics, molecular characterisation, and vaccine development for rickettsial diseases depend on the *in vitro* isolation of viable pathogens. As obligate intracellular bacteria, *O. tsutsugamushi* and *Rickettsia* spp. require specialised culture techniques, biosafety facilities, and expert handling. At a time when regional capacity for rickettsial culture was limited, a reference platform for *in vitro* isolation was established to support diagnostics, genomics, and pathogenesis studies.

### 5.1 Building *in vitro* culture capacity

The first *O. tsutsugamushi* isolates obtained in Thailand in the 2000s were recovered from fever patients in collaboration with Udon Thani Hospital, northeastern Thailand [116] and the Shoklo Medical Research Unit (SMRU) on the Thai-Myanmar border [12,117,118] between 2004 and 2006 at the Mahidol Oxford Tropical Medicine Research Unit (MORU) in Bangkok. *In vitro* isolation was attempted in Vero cells, and *O. tsutsugamushi* was isolated from 12 samples (sensitivity 46.7%), with a time to isolation of 16–37 days (median 27 days) [117]. *In vitro* cultures continue to be obtained from clinical samples of patients with fever in Chiang Rai and Mae Sot (i.e., SMRU), Thailand, to the present day.

From 2008 to 2014, the Lao-Oxford-Mahosot Hospital-Wellcome Trust Research Unit (LOMWRU) and MORU-Bangkok expanded *in vitro* isolation efforts in Thailand and Laos, processing over 3,200 patient blood samples and establishing one of the largest regional collections of *O. tsutsugamushi* and *R. typhi* isolates in Southeast Asia [119]. This bank of *in vitro* isolates has supported the validation of serological assays, molecular and antigenic characterisation, and preclinical vaccine development, while also generating detailed data on determinants of culture success. At Mahosot Hospital in Vientiane, overall isolation success was 7.9%, rising to 17.3% among patients with positive rapid diagnostic tests, serology, or PCR results. Isolation success was strongly seasonal, peaking at 28.3% in November, and was associated with longer illness duration, positive quantitative PCR, the absence of prolonged antibiotic use, buffy coat inoculation, and shorter delays between sample collection and culture.

### 5.2 Optimising culture and storage methods

Subsequent studies optimised protocols for yield, purification, and cryopreservation [120]. Building on these, further optimisation informed best practices for handling and storing rickettsial isolates. These improvements maintained long-term viability and preserved antigenic and genomic integrity, while passaging protocols minimised genetic drift. These improvements help strains retain their diagnostic accuracy and viability for applications such as ELISA and genome sequencing. The isolate bank underpins ongoing research in diagnostics and genomics, supporting standardisation, surveillance, and vaccine development.

### 5.3 Extending culture capacity into one health investigations

While early *in vitro* isolation focussed on human clinical specimens, subsequent work showed that culture platforms could be extended to outbreak and ecological investigations. Following a scrub typhus outbreak at a military training base in central Thailand, *O. tsutsugamushi* was successfully isolated from rodents captured in the affected area, directly linking human cases to local animal reservoirs [121]. This demonstrated that viable *O. tsutsugamushi* could be recovered from field-collected wildlife under operational conditions, thereby enabling the integration of clinical surveillance, field ecology, and laboratory science. Importantly, animal-derived isolates were incorporated into the same culture and banking pipelines as human strains, enabling comparative genomic, antigenic, and diagnostic studies. Together, this work established *in vitro* isolation as a core enabling technology for One Health rickettsiology, underpinning outbreak investigation, ecological inference, and translational research in endemic settings.

## 6. Genetic and antigenic characterisation

Understanding the genetic and antigenic diversity of *O. tsutsugamushi* has been vital to addressing persistent challenges in diagnosis, surveillance, and vaccine development. These studies have highlighted the heterogeneity of *O. tsutsugamushi* and its implications for diagnostics and control strategies.

### 6.1 Single-gene diversity studies

Initial molecular characterisation of *O. tsutsugamushi* focussed on the 56-kDa type-specific antigen (TSA) gene, the principal target of most diagnostic and serological assays. Sequencing of clinical isolates from Thailand and Laos demonstrated extensive genetic heterogeneity within and between sites, challenging the assumption that a single antigenic target could adequately represent regional diversity [122,123] (Table 3). Among 23 isolates obtained from patients in north-eastern (Udon Thani) and western Thailand between 2003 and 2005, multiple genotypes were identified, including Karp-, Gilliam-, TA716-, and TA763-like strains, with considerable sequence diversity comparable to global strain variation [122]. More recent molecular surveillance in Chiang Rai and Mae Sot in northern Thailand confirmed continued circulation of diverse strains, including Karp-, Gilliam-, Taiwan-, P23-, and CM606-like genotypes, with eschar-derived PCR demonstrating the highest diagnostic sensitivity [107]. Similarly, a genotyping study in northern Vietnam identified substantial diversity among PCR-confirmed cases, with Karp predominating (55%) alongside TA763, Gilliam (Japan variant), and Kato strains [124]. Parallel analysis of the 47-kDa *htrA* gene in human isolates further demonstrated marked sequence variability, indicating that antigenic heterogeneity extends beyond the TSA locus [125]. At the ecological level, rodent surveillance by Armed Forces Research Institute of Medical Sciences (AFRIMS) in Bangkok, Thailand, yielded multiple novel isolates, some unique to specific local transmission foci [121], reinforcing evidence of ongoing diversification in enzootic reservoirs. Investigation of *O. tsutsugamushi*-positive larval trombiculid mites collected from small mammals across Thailand revealed marked heterogeneity in genotypes, as determined by sequencing of the 56kDa gene [126]. The presence of multiple *O*. *tsutsugamushi* genotypes within individual mites raises the possibility that co-feeding transmission may contribute to pathogen diversification, consistent with evidence of extensive recombination in *O. tsutsugamushi* populations [126,127]. A nationwide human study in Thailand further demonstrated pronounced spatial structuring of genotypes, with distinct strain clusters dominating different ecological zones and substantial heterogeneity even within provinces [128].

**Table 3:** Single-gene (56-kDa TSA/ 47-kDa *htrA*) diversity of *O. tsutsugamushi*—Thailand, Vietnam and Laos.

Country/Province/Region	Studies	Sample type (year)	Gene target	Identified strain types/Notes
Thailand—Udon Thani (NE)	[122]	Clinical isolates (2003–2005)	56-kDa (full ORF)	Karp (15/23), Gilliam (6/23), TA716-like, TA763-like
Thailand—Tak Province (W)	[122]	Clinical isolates (2003–2005)	56-kDa	Similar distribution to Udon Thani; Karp & Gilliam prominent
Thailand—Chiang Rai (N)	[107]	Buffy coat & eschar (2018–2019)	56-kDa (partial)	Karp, Gilliam, Taiwan-like, P23-like, CM606-like
Thailand—Mae Sot (N, border with Myanmar)	[107]	Human isolates (2018–2019)	56-kDa	Karp, Gilliam, P23-like, CM606-like
Thailand—Nationwide (multiple provinces)	[128]	Human isolates (nationwide sampling)	56-kDa	Pronounced spatial structuring; province-level clusters of Karp, Gilliam, TA763-like, and related variants
Thailand—Chonburi (AFRIMS rodent surveillance)	[121]	Rodent isolates	56-kDa	Six novel isolates; unique or closely related to Thai genotypes (local enzootic diversity)
Vietnam—Northern Vietnam	[125]	Clinical cases (*n* = 63; PCR+ 42)	56-kDa (partial)	Karp predominates (~55% of PCR+), plus TA763, Gilliam (Japan variant), Kato
Laos—multiple sites	[122,123]	Clinical isolates/surveillance	56-kDa (and other loci)	Extensive genetic heterogeneity reported across sampled sites; specific dominant genotypes not specified in summary
Human isolates (multiple Southeast Asian sites)	[107,125]	Human clinical/eschar samples	47-kDa *htrA*	High sequence variability across isolates; antigenic heterogeneity extends beyond 56-kDa TSA

Collectively, these findings demonstrate a highly heterogeneous pathogen population across Southeast Asia and provide a mechanistic explanation for the inconsistent performance of single-antigen diagnostic assays, supporting the need for multivalent diagnostic platforms and vaccine strategies that account for substantial geographic turnover and local strain diversity [129–131].

### 6.2 Whole-genome sequencing and structural genomics

To explore the genomic diversity of *O. tsutsugamushi* beyond single-gene analysis, studies employed multilocus sequence typing (MLST) and long-read whole-genome sequencing of locally cultivated isolates. These methods provided a high-resolution view of genetic variation, recombination, and genome structure. These efforts uncovered exceptionally high rates of homologous recombination, resulting in extensive intra-species genetic variability [127]. MLST analyses of isolates from Cambodia, Vietnam and Thailand further demonstrated that, despite strong geographic clustering observed in the highly variable 56-kDa antigen gene, the core genome exhibits weak geographic structuring and forms a single regional metapopulation, with ancestral haplotypes maintained through ongoing recombination in mite vectors [132]. MLST studies of 74 clinical *O. tsutsugamushi* isolates from three Lao locations, compared with isolates from Udon Thani, northeast Thailand, demonstrated high diversity and recombination in the population, with ~8% mixed infection [133]. In the same study, there was low population differentiation between Vientiane and Udon Thani, on opposite sides of the Mekong River, but a distinct population in Salavan, southern Laos [133].

Whole-genome sequencing of Thai *O. tsutsugamushi* isolates yielded six complete genomes, providing the first detailed insights into the structure of Southeast Asian strains. Further analysis revealed a small core genome (~657 genes), embedded within highly repetitive, fragmented regions, complicating assembly and comparative genomics [134]. The discovery of the organism’s genomic plasticity was striking, with abundant mobile genetic elements, gene duplications, and pseudogenes contributing to low synteny across strains, even within similar areas [134]. The study also found important discrepancies between 56kDa and whole-genome genotyping, highlighting the need for a better understanding of relatedness among strains. Target enrichment approaches have shown promise, enabling the generation of partial *O. tsutsugamushi* sequences from mites and rats to investigate phylogeographic clustering across host species [124] and to facilitate sequencing of clinical isolates of *O. tsutsugamushi* and *R. prowazekii* [135]. Nanopore sequencing of an *R. typhi* isolate in Lao PDR demonstrated that MinION data can generate complete genomes in resource-limited settings and was followed by a larger study comparing recent isolates from Laos with a historical collection [136]. These data represent the first complete genome sequences of *R. typhi* isolates collected over the last 50 years and demonstrate extremely low genetic diversity across the species over a 90-year timespan, a stark contrast to the complex genetics of *O. tsutsugamushi* [137].

### 6.3 Antigenic cartography

Antigenic cartography, adapted from influenza research, was used to visualise cross-reactive relationships among scrub typhus strains based on IFA data from Thai isolates [138]. It showed extensive cross-reactivity, with antigenic distances often not correlating with genetic ones, highlighting limitations of sequence data alone. The approach emphasises the need for multivalent or geographic-specific antigen panels to enhance serology-based diagnostics.

## 7. Redefining the pathogenesis of rickettsial infections

Historically, the pathogenesis of scrub typhus and murine typhus was poorly understood, with limited data on cellular targets, immune responses, or pathogen-specific mechanisms of rickettsial pathogenesis. Key findings link clinical phenotypes to immune pathways, overturn assumptions about cellular targets, and support vaccine development through novel animal models. These advances were supported by long-standing regional partnerships, including AFRIMS in Bangkok, which has played a central role in animal models, vector biology, and translational immunology for rickettsial diseases in Southeast Asia.

### 7.1 Comparative human immunopathology and experimental models

Comparative clinical studies in Laos and Thailand first demonstrated that scrub typhus and murine typhus elicit distinct immune response profiles in endemic populations. Biomarker analyses showed stronger endothelial activation in murine typhus, whereas scrub typhus was characterised by heightened monocyte and leukocyte activation, with divergent coagulation and inflammatory pathways closely linked to disease severity [139,140]. These differences align with clinical patterns: murine typhus usually presents as a milder, viral-like illness (although CNS manifestations, such as reduced consciousness, can occur), while scrub typhus is more likely to cause intense inflammation and multi-organ involvement. The isolation of local *O. tsutsugamushi* strains enabled the development of more physiologically relevant infection models. Intradermal infection of Swiss CD-1 mice demonstrated that virulent strains disseminate more rapidly than less pathogenic isolates, establishing a mechanistic link between strain variation and clinical severity [141]. This approach captured early host–pathogen interactions at the dermal entry site, advancing pathogenesis research beyond artificial intravenous models.

Building on this work, non-human primate models using rhesus and cynomolgus macaques reproduced human-like disease, systemic dissemination, and robust cellular immune responses, providing platforms for immunopathogenesis and vaccine evaluation [142–144]. Comparative macaque studies further demonstrated strain-specific differences in clinical progression and immune responses, informing antigen selection for vaccine development [145]. Histopathological studies of human eschars suggest that early infection is characterised by predominant infection of dendritic cells and monocytes, rather than endothelial cells, challenging the traditional view of primary endothelial tropism [146].

### 7.2 Vector ecology

Vector ecology has been central to understanding scrub typhus transmission in Southeast Asia. The disease is maintained in enzootic cycles involving trombiculid mite larvae (chiggers), small mammals, and ecological niches, especially but not exclusively secondary vegetation, forest fringes, and agricultural land. Experimental work, including the establishment and maintenance of a laboratory chigger colony at the Armed Forces Research Institute of Medical Sciences (AFRIMS) in Thailand, has been critical for elucidating vector competence, transovarial transmission, and strain diversity of *O. tsutsugamushi*, providing foundational insights into transmission dynamics [5,147]. Advances in mite taxonomic identification using autofluorescence microscopy and mitochondrial *coi* gene sequencing have been critical in advancing this neglected and challenging area of vector ecology for scrub typhus [148].

In Northern Thailand, field studies have identified habitat complexity and ecotones as important determinants of scrub typhus [149–151]. High rodent density in fragmented habitats likely supports the maintenance of vector species of chigger; the factors that influence risk of scrub typhus infection at different scales remain poorly understood [150]. Human behaviour substantially modifies exposure risk. In Northern Thailand, studies have shown that agricultural practices, forest-related activities, and ground-level occupational exposure increase the likelihood of contact with infected chigger habitats. At the same time, limited awareness and delayed healthcare-seeking contribute to underdiagnosis [49,150]. Together, these findings emphasise that rickettsial transmission in Southeast Asia is shaped by the interaction between vector ecology and socio-behavioural factors, reinforcing the need for integrated ecological surveillance and community-level prevention strategies.

## 8. Treatment and intervention studies

Collaborative studies strengthen impact by linking laboratory research with clinical practice. Integration of improved diagnostics with prospective clinical studies has enabled randomised controlled trials defining optimal antimicrobial treatment for rickettsial infections. These findings have directly informed regional treatment protocols and international guidelines for the management of febrile illness.

### 8.1 Randomised controlled trials (RCTs)

Randomised controlled trials have provided stronger evidence for the treatment of rickettsial diseases, increasing confidence in clinical management across endemic settings. In Thailand, a randomised trial comparing a 7-day course of doxycycline with a 3-day course of azithromycin demonstrated that azithromycin was non-inferior for the treatment of scrub typhus and leptospirosis, with similar fever clearance times and fewer adverse events. However, doxycycline remained the more affordable option [152]. In Laos, a randomised trial at Mahosot Hospital showed that, in contrast, for uncomplicated murine typhus, although 3 days and 7 days of oral doxycycline were safe and efficacious, 3 days of azithromycin were inferior in terms of fever clearance time [153]. The reason why azithromycin has inferior efficacy to doxycycline in murine typhus but not in scrub typhus remains to be determined. Also, the optimal duration of doxycycline treatment in uncomplicated scrub typhus remains to be determined. For severe scrub typhus, a multicentre trial conducted by the Christian Medical College, Vellore, across multiple sites in India demonstrated that while intravenous doxycycline or azithromycin monotherapy was effective and well tolerated, combination therapy was a superior therapeutic option [154]. Together, these trials directly informed treatment guidelines for severe scrub typhus across Asia and improved clinical care pathways for rickettsial infections in endemic and resource-limited settings.

### 8.2 Antimicrobial resistance

Reports of reduced clinical responsiveness to doxycycline in scrub typhus were first described in northern Thailand in the 1990s, with documented delayed fever clearance times suggesting possible antimicrobial resistance in *O. tsutsugamushi* isolates from Chiang Rai Province [155,156]. These observations prompted concern regarding emerging doxycycline resistance in Southeast Asia. Subsequent laboratory investigations, including cell culture–based susceptibility testing of Thai and Lao isolates, did not demonstrate high-level resistance but did reveal variability in *in vitro* susceptibility profiles [157]. More recent re-examination of clinical and microbiological evidence from Thailand & Lao concluded that convincing evidence of stable doxycycline resistance is lacking, with delayed clinical responses more likely attributable to host and pathogen strain diversity, or pharmacokinetic factors, rather than to true antimicrobial resistance [158]. However, based on the genomes of reference and Lao strains, it was determined that *O. tsutsugamushi* would be intrinsically resistant to fluoroquinolones *in vivo* [116]*.* This class of antibiotics, as well as cotrimoxazole, should not be used for the treatment of scrub typhus. Collectively, current evidence supports the continued use of doxycycline as the first-line therapy for scrub typhus in Southeast Asia, while highlighting the importance of ongoing clinical and molecular surveillance.

### 8.3 Rickettsial infections in pregnancy

Studies along the Thai–Myanmar border, led by the SMRU, identified scrub typhus and murine typhus as major, under-recognised threats to maternal and neonatal health, and found that they were often misdiagnosed or untreated due to limited diagnostic capacity. Foetal outcomes were also adversely affected, with increased rates of miscarriage and stillbirth observed among infected women, particularly when maternal diagnosis was delayed or missed [72]. This study was among the first prospective investigations worldwide to quantify the maternal and perinatal burden of rickettsial diseases. Scrub typhus in pregnancy presents a major diagnostic and therapeutic dilemma in resource-limited endemic settings. Case reports and cohort data from Southeast Asia demonstrate high rates of adverse maternal and foetal outcomes, including miscarriage, stillbirth, preterm birth, and low birth weight, with poor neonatal outcomes reported in up to 36%–42% of cases [72]. In a case report from the Thai–Myanmar border, chloramphenicol treatment was associated with maternal survival but not foetal survival, highlighting the difficult risk–benefit decisions required when managing severe disease [159]. Evidence supporting azithromycin, the most commonly used alternative in pregnancy, remains limited, and azithromycin is likely to have limited efficacy in murine typhus. Fever clearance time appears associated with pregnancy outcome, underscoring the urgent need for prospective trials and context-appropriate treatment guidelines in endemic areas. The evidence regarding the safety of doxycycline use during pregnancy for treating rickettsial infections has also been reviewed [160]. Given the adverse outcomes associated with scrub typhus in pregnancy, and the increasing evidence of its safety in pregnancy, doxycycline is the drug of choice.

### 8.4 Community engagement

Despite increased recognition of rickettsial diseases as a major infectious cause of fever in the region, they remain largely ignored by the public and, often, by healthcare workers. Recently, in northern Thailand, a community engagement project promoted disease prevention and successfully increased knowledge and awareness of scrub typhus among at-risk populations [161].

## 9. Summary of impacts and future directions

Over two decades of integrated rickettsial research across Southeast Asia, our understanding of these infections has been fundamentally reshaping how they are diagnosed and managed. Through global and regional collaborations (see Table 4), this body of work has reframed scrub typhus, murine typhus, and related rickettsioses from obscure academic entities into core components of febrile illness policy, surveillance, and clinical care across multiple health systems (see Panel 1 and Table 5 for summary).

**Table 4:** Major research collaborations and institutional networks contributing to rickettsial disease research in Southeast Asia. This table highlights key groups and partnerships and is not intended to represent an exhaustive list of all contributing institutions.

Partner institution/network	Location	Type of collaboration	Primary contributions
Mahidol Oxford Tropical Medicine Research Unit (MORU)	Bangkok, Thailand	Oxford NDM Overseas Research Unit	Study leadership; diagnostics development; epidemiology; culture, genomics, pathogenesis; clinical trials; One Health integration
Oxford University Clinical Research Unit (OUCRU)	Vietnam, Indonesia	Oxford NDM Overseas Research Unit	Seroepidemiology, historical aspects
Centre for Tropical Medicine and Global Health, Nuffield Department of Medicine, University of Oxford (Oxford NDM)	Oxford, UK	Parent academic institution	Academic governance; Strategic oversight
Lao-Oxford-Mahosot Hospital-Wellcome Trust Research Unit (LOMWRU)	Vientiane, Laos	MORU Network Unit	Febrile illness surveillance; CNS infection studies; serology; molecular diagnostics; *in vitro* isolation; ecological studies; murine typhus RCT
Shoklo Malaria Research Unit (SMRU)	Mae Sot	MORU Network Unit	Pregnancy cohorts; scrub & murine typhus in pregnancy; diagnostics; treatment studies
Cambodia-Oxford Medical Research Unit (COMRU)	Siem Reap, Cambodia	MORU Network Unit	Paediatric febrile illness surveillance; hospital-based epidemiology; diagnostics
Myanmar–Oxford Clinical Research Unit (MOCRU)	Myanmar	MORU Network Unit	Seroepidemiology; febrile illness studies
Chiang Rai Clinical Research Unit (CCRU)	Northern Thailand	MORU network site	Adult and paediatric febrile illness studies; scrub typhus severity; landscape epidemiology
Udon Thani Hospital	Northeastern Thailand	MORU clinical site	Early human isolate collection; *in vitro* culture development
Christian Medical College (CMC)	Vellore, India	International clinical research partner	Severe scrub typhus RCTs; epidemiology; diagnostics validation
Chittagong Medical College Hospital	Chittagong, Bangladesh	International clinical research partner	Febrile illness studies; scrub and murine typhus burden; serological validation
Facultad de Medicina Clínica Alemana, Universidad del Desarrollo	Santiago, Chile	International clinical research partner	Characterisation of *Candidatus* Orientia chiloensis
Australian Rickettsial Reference Laboratory (ARRL)	Geelong, Australia	International reference laboratory	*In vitro* culture; molecular characterisation; characterisation of *Candidatus* Orientia chuto
Centre National de Référence Rickettsia	Marseille, France	International reference laboratory	Serology; global rickettsiology expertise
Armed Forces Research Institute of Medical Sciences (AFRIMS)	Bangkok, Thailand	International clinical research partner	Non-human primate studies
US Navy—Naval Medical Research Center	Silver Spring, USA	International reference laboratory	Serology; global rickettsiology expertise
University of Texas Medical Branch	Galveston, USA	International clinical research partner	Global rickettsiology expertise
Rickettsia Threat Reduction Network (DTRA-funded)	Asia-Pacific	Regional capacity-building and harmonisation network	Laboratory harmonisation; biosafety; molecular & serological diagnostics; training

**Table 5:** Key scientific contributions and public health impact of long-term rickettsial research in Asia.

Domain	Key contributions	Impact
Epidemiology & Surveillance	Multi-country studies identifying scrub and murine typhus as leading febrile causes	Reframed public health understanding; displaced malaria-centric assumptions
	UFI studies across Laos, Thailand, Myanmar, India, Bangladesh, Nepal, Cambodia, Indonesia	Enabled national recognition and prioritisation of rickettsial diseases
	SEACTN and One Health studies linking ecology to disease	Embedded rickettsioses into land-use and climate health frameworks
Diagnostics	Standardisation of IFA/ELISA cut-offs using Bayesian modelling and kinetics data	Improved diagnostic reliability; reduced false positives/negatives
	Evaluations of RDTs and LAMP PCR assays	Facilitated field-level diagnosis in low-resource settings
	Systematic reviews of diagnostic performance	Shaped regional diagnostic policy and laboratory standards
*In Vitro* Culture	Over 3,200 blood cultures from Thailand and Laos; sensittivity 46.7% (Thai) and 7.9% (Lao)	Created isolate banks for diagnostics, genomics, and vaccine research
	Optimisation of storage and propagation methods	Sustained viability and research usability of strains
Genomics & Antigenic Diversity	56kDa/47kDa gene studies and whole genome sequencing	Revealed extreme genetic variability and recombination
	Antigenic cartography of Thai isolates	Guided design of diagnostic antigen panels and vaccine selection
Pathogenesis & Immunology	Comparative cytokine and endothelial profiling in human cases	Explained clinical differences between scrub and murine typhus
	Discovery of dendritic/monocyte tropism	Overturned endothelial tropism dogma
	Macaque model development and vaccine challenge studies	Defined immune correlates of protection for vaccine research and development
Treatment Studies	Documented maternal and foetal risk in rickettsial pregnancy	Informed clinical guidelines for management in pregnancy
	RCTs on doxycycline and azithromycin for murine and scrub typhus	Defined evidence-based treatments for mild and severe cases

UFI, Undifferentiated Febrile Illness; SEACTN, Southeast Asia Clinical Trials Network; IFA, Indirect Immunofluorescence Assay; ELISA, Enzyme-Linked Immunosorbent Assay; RDTs, Rapid Diagnostic Tests; LAMP PCR, Loop-Mediated Isothermal Amplification Polymerase Chain Reaction; PCR, Polymerase Chain Reaction; 56 kDa/ 47 kDa, 56-kilodalton and 47-kilodalton antigen genes; RCTs, Randomised Controlled Trials.

**Fig 1:**
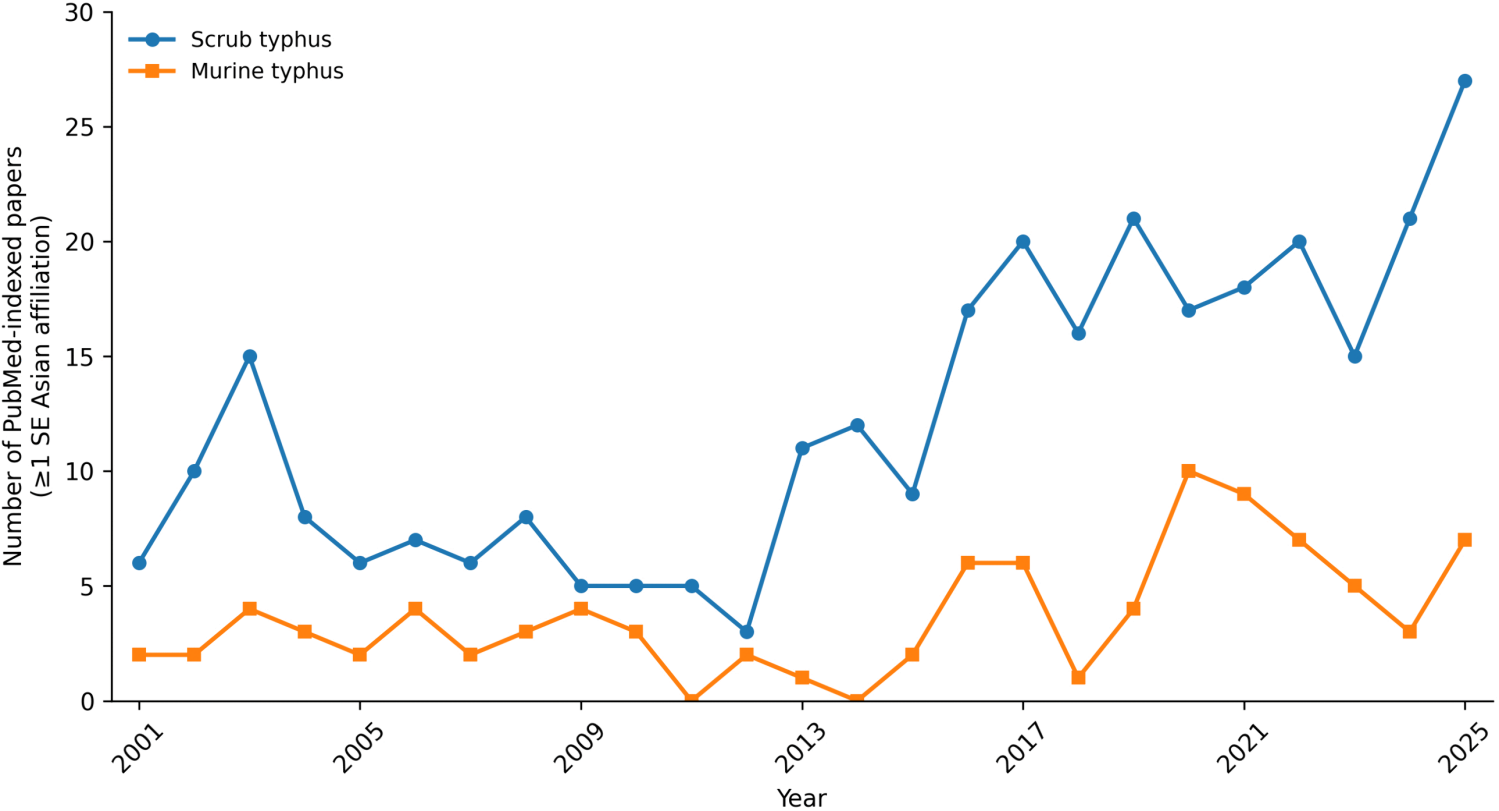
Annual number of PubMed-indexed publications on scrub typhus and murine typhus with at least one author affiliated to a Southeast Asian institution, 2001–2025.

Between 2001 and 2025, 318 publications on scrub typhus and 97 on murine typhus were identified from Southeast Asian institutions (Fig 1). Scrub typhus output increased markedly after 2012, peaking at 27 publications in 2025, whereas murine typhus publications remained comparatively lower, with a maximum annual output of 10 in 2019. The sustained increase in publications on scrub typhus from Southeast Asian institutions, with more than half (55.0%) involving affiliations with MORU and LOMWRU, reflects not only the regional disease burden but also institutional leadership and funding. The MORU network units in Thailand and Laos have been identified as the most productive global institution in scrub typhus research [162], and diagnostic-focussed bibliometric analyses further confirm their central role in advancing laboratory methodologies for rickettsial diseases [163].

The impact of these studies goes beyond publications. Epidemiological platforms have shown that rickettsial infections account for 10%–25% of acute febrile admissions, challenging the idea that malaria and viral infections are the primary drivers of fever. Diagnostic research built the first regional architecture for rickettsial serology and testing in Asia, fixing decades of inconsistent case definitions and enabling reliable burden estimates. Culture and isolate banking developed regional capacity for pathogen analysis, assay validation, and translational research, skills once limited to a few high-income laboratories. Genomic studies explained why many diagnostics fail in endemic areas, guiding the design of better, multivalent assays. All these efforts created a translational pipeline from ecology to treatment, policy, and preparedness. This integrated system sets the programme apart from traditional research, showing how sustained investment can turn fragmented studies into lasting public health infrastructure.

Several lessons emerge. First, pathogen-agnostic febrile illness surveillance is vital for detecting under-recognised causes of severe disease in settings with limited diagnostics. Second, diagnostic strategies must be tailored to local epidemiology and distinguish between tools for patient management and those for surveillance and policy. Third, empirical treatment, like early doxycycline in suitable cases, remains a practical, evidence-based approach to prevent morbidity and mortality as diagnostics improve. Fourth, local collaborative research agenda decision-making allows responsiveness to key but neglected local research questions. Lastly, these gains require sustained lab platforms, harmonised methods, and a trained regional workforce, not short-term projects.

The identification and validation of prognostic biomarkers represent an important future direction for improving clinical management of rickettsial diseases. Studies from Southeast Asia have demonstrated associations between host immune responses, endothelial activation, and disease severity, suggesting that biomarker-based risk stratification may be feasible. Integration of such biomarkers into clinical algorithms could enable early identification of patients at risk of severe disease, guide triage decisions, and optimise resource allocation. However, further validation across diverse populations and translation into affordable, point-of-care formats will be essential before routine implementation.

This evidence base has limitations. Much of the data comes from sentinel sites with better diagnostic capabilities, so they are not nationally representative. Diagnostic thresholds, assays, and case definitions vary, complicating comparisons. Many Southeast Asian countries are underrepresented in surveillance, with only Thailand having a mandatory national surveillance system. Still, consistent findings across diverse methods and populations support the conclusion that rickettsial diseases are common, often under-recognised, clinically significant, and respond well to low-cost interventions when health systems are well coordinated.

Looking ahead, Southeast Asia faces rising risks from vector-borne and zoonotic infections due to climate change, land use change, urbanisation, and population mobility [24]. The strategies here, integrated fever surveillance, calibrated diagnostics, regional labs, and clinical trials, provide a transferable preparedness framework for endemic and emerging pathogens. However, these capabilities are fragile. Sentinel platforms decline without funding; calibration standards drift; *in vitro* isolate collections and molecular capacity weaken or disappear; and health systems revert to reactive fever management. Recent research on anti-rickettsial vaccines in Southeast Asia is extremely limited, but renewed efforts, especially for scrub typhus, could improve prevention [130,131].

Emerging technologies, including artificial intelligence (AI)-supported diagnostics and digital surveillance systems, offer important opportunities to strengthen rickettsial disease detection and reporting in Southeast Asia. AI-based approaches could support the integration of clinical, laboratory, and epidemiological data to improve diagnostic accuracy in settings where confirmatory testing is limited. In parallel, expansion of national surveillance systems, building on existing sentinel platforms, is feasible and would substantially improve burden estimation, outbreak detection, and public health response. However, implementation will depend on sustained investment in digital infrastructure, data standardisation, and integration with existing health systems. Experience from Thailand, where scrub typhus is notifiable, demonstrates that scalable surveillance is achievable, although broader regional adoption remains limited.

Collectively, this synthesis provides new perspectives by linking long-term epidemiological trends, diagnostic advances, and clinical trial evidence into a coherent regional framework. This integrated view highlights how sustained investment in collaborative research platforms can directly translate into improved patient management and more effective public health strategies for neglected febrile illnesses.

The past 25 years have taught a key lesson: progress against neglected infectious diseases depends on long-term investment in health systems, not just on studies with dedicated funding. Maintaining regional frameworks enables early detection of ecological shifts, quick validation of diagnostics, and real-time data integration. The recent development of the Asia-Pacific Rickettsial Conferences has facilitated liaison and a more coordinated approach. Southeast Asia’s rickettsial research infrastructure offers a foundation for broader epidemic preparedness. Despite high disease burdens, especially for scrub typhus, funding remains limited to address these neglected issues and shape policy. This review shows that with sustained support, endemic regions can produce world-class science, inform policy, and build resilient health systems. The future focus should be on preserving, strengthening, and scaling these platforms.

Panel 1. Implications for febrile illness policy in Southeast Asia. These points synthesise key insights derived from 25 years of integrated epidemiological, clinical, diagnostic, and translational research across Southeast Asia, highlighting how sustained regional collaboration has transformed rickettsial diseases from under-recognised causes of fever into actionable priorities for clinical management and public health policy.

- **Rickettsial diseases should be explicitly incorporated into national undifferentiated febrile illness (UFI) guidelines**, particularly in rural and peri-urban settings where access to diagnostic services is limited.
- **Empiric doxycycline therapy should be considered early** for patients with compatible clinical syndromes, especially CNS infections, given its low cost, safety profile, and proven mortality benefit.
- **Reliance on rapid serology-based diagnostic tests alone is insufficient**; calibrated serological and molecular diagnostics, ideally combined at the point of care, remain essential for surveillance and outbreak detection.
- **Sentinel surveillance platforms**, including hospital-based systems, provide cost-effective approaches to monitoring rickettsial transmission in endemic regions. Ensure that scrub typhus is a nationally reportable infection.
- **Regional laboratory harmonisation and training networks** are feasible and critical for ensuring diagnostic comparability, outbreak preparedness, rapid pathogen identification, and locally appropriate diagnostic cut-offs.
- **Integrated One Health approaches** linking human, animal, and environmental surveillance will strengthen preparedness for emerging and re-emerging vector-borne diseases.

Key learning points, key papers, and advances in rickettsial diagnostics in Southeast Asia

**Learning points**

- Rickettsial infections are major but persistently under-recognised causes of acute febrile illness in Southeast Asia, frequently accounting for 10%–25% of hospitalised cases.
- Diagnostic limitations, particularly reliance on poorly standardised serology, have historically led to underestimation of disease burden and misclassification of cases.
- Locally validated serological cut-offs and integration of molecular diagnostics have substantially improved detection, surveillance, and clinical management.
- Early empiric treatment with doxycycline remains highly effective and is critical for reducing morbidity and mortality in endemic settings.
- Long-term regional investment in laboratory platforms, surveillance systems, and collaborative research networks can transform neglected diseases into integrated public health priorities.

Key papers in the field

- Mayxay M, Castonguay-Vanier J, Chansamouth V, Dubot-Peres A, Paris DH, Phetsouvanh R, et al. Causes of non-malarial fever in Laos: a prospective study. Lancet Glob Health. 2013;1(1):e46-54. https://doi.org/10.1016/S2214-109X(13)70008-1. PubMed PMID: 24748368; PubMed Central PMCID: PMCPMC3986032.Dittrich S et al. *CNS infections in Laos*. Lancet Glob Health. 2015.
- Phongmany S, Rolain JM, Phetsouvanh R, Blacksell SD, Soukkhaseum V, Rasachack B, et al. Rickettsial infections and fever, Vientiane, Laos. Emerg Infect Dis. 2006;12(2):256–62. https://doi.org/10.3201/eid1202.050900. PubMed PMID: 16494751; PubMed Central PMCID: PMCPMC3373100.
- Varghese GM, Dayanand D, Gunasekaran K, Kundu D, Wyawahare M, Sharma N, et al. Intravenous Doxycycline, Azithromycin, or Both for Severe Scrub Typhus. N Engl J Med. 2023;388(9):792–803. https://doi.org/10.1056/NEJMoa2208449. PubMed PMID: 36856615; PubMed Central PMCID: PMCPMC7614458.
- Blacksell SD, Bryant NJ, Paris DH, Doust JA, Sakoda Y, Day NP. Scrub typhus serologic testing with the indirect immunofluorescence method as a diagnostic gold standard: a lack of consensus leads to a lot of confusion. Clin Infect Dis. 2007;44(3):391–401. Epub 20070103. https://doi.org/10.1086/510585. PubMed PMID: 17205447.
- Batty EM, Chaemchuen S, Blacksell S, Richards AL, Paris D, Bowden R, et al. Long-read whole genome sequencing and comparative analysis of six strains of the human pathogen *Orientia tsutsugamushi*. PLoS Negl Trop Dis. 2018;12(6):e0006566. Epub 20180606. https://doi.org/10.1371/journal.pntd.0006566. PubMed PMID: 29874223; PubMed Central PMCID: PMCPMC6005640.

Advantages of emerging diagnostic technologies

- Molecular assays (PCR, LAMP) enable early detection during the acute phase when serology is often negative.
- Multiplex and quantitative PCR platforms allow simultaneous detection and differentiation of co-circulating rickettsial pathogens.
- Integration of molecular, serological, and rapid tests provides a layered diagnostic framework adaptable to different clinical and surveillance settings.

Disadvantages of emerging diagnostic technologies

- Diagnostic performance is highly dependent on local validation, including strain diversity and appropriate serological cut-offs.
- Molecular diagnostics require infrastructure, training, and quality assurance systems that remain limited in many endemic settings.
- Rapid diagnostic tests show variable sensitivity and specificity, limiting their reliability for standalone clinical decision-making.
